# Transcriptomic and Metabolic Network Analysis of Metabolic Reprogramming and IGF-1 Modulation in SCA3 Transgenic Mice

**DOI:** 10.3390/ijms22157974

**Published:** 2021-07-26

**Authors:** Yu-Te Lin, Yong-Shiou Lin, Wen-Ling Cheng, Jui-Chih Chang, Yi-Chun Chao, Chin-San Liu, An-Chi Wei

**Affiliations:** 1 Graduate Institute of Biomedical Electronics and Bioinformatics, National Taiwan University, Taipei 10617, Taiwan; F06945036@ntu.edu.tw; 2 Institute of ATP, Vascular and Genomic Center, Changhua Christian Hospital, Changhua 50091, Taiwan; 398157@cch.org.tw (Y.-S.L.); 111800@cch.org.tw (W.-L.C.); 145520@cch.org.tw (J.-C.C.); 3 Inflammation Research & Drug Development Center, Changhua Christian Hospital, Changhua 50091, Taiwan; 183782@cch.org.tw; 4 Department of Neurology, Changhua Christian Hospital, Changhua 50091, Taiwan; 5 Graduate Institute of Integrated Medicine College of Chinese Medicine, China Medical University, Taichung 40402, Taiwan; 6 Department of Electrical Engineering, National Taiwan University, Taipei 10617, Taiwan

**Keywords:** spinocerebellar ataxia type 3, insulin-like growth factor 1, RNA-seq, context-specific metabolic networks

## Abstract

Spinocerebellar ataxia type 3 (SCA3) is a genetic neurodegenerative disease for which a cure is still needed. Growth hormone (GH) therapy has shown positive effects on the exercise behavior of mice with cerebellar atrophy, retains more Purkinje cells, and exhibits less DNA damage after GH intervention. Insulin-like growth factor 1 (IGF-1) is the downstream mediator of GH that participates in signaling and metabolic regulation for cell growth and modulation pathways, including SCA3-affected pathways. However, the underlying therapeutic mechanisms of GH or IGF-1 in SCA3 are not fully understood. In the present study, tissue-specific genome-scale metabolic network models for SCA3 transgenic mice were proposed based on RNA-seq. An integrative transcriptomic and metabolic network analysis of a SCA3 transgenic mouse model revealed that metabolic signaling pathways were activated to compensate for the metabolic remodeling caused by SCA3 genetic modifications. The effect of IGF-1 intervention on the pathology and balance of SCA3 disease was also explored. IGF-1 has been shown to invoke signaling pathways and improve mitochondrial function and glycolysis pathways to restore cellular functions. As one of the downregulated factors in SCA3 transgenic mice, IGF-1 could be a potential biomarker and therapeutic target.

## 1. Introduction

Spinocerebellar ataxia (SCA) is a neurodegenerative genetic disease characterized by common clinical features such as gait instability and affects motor coordination, resulting in alterations in limb control, language, and eye movements. SCA has been classified into dozens of different subtypes, among which spinocerebellar ataxia type 3 (SCA3) is one of the most common in Asia. SCA3 is a polyglutamine neurodegenerative disease resulting from abnormal CAG triplet repeats in the ATXN3 gene (14q21) that lead to the misfolding and accumulation of a pathogenic protein, causing cerebellar dysfunction [1].

SCA3 is still considered a continuously progressing and irreversible disease with no effective treatment. The current main principles of treatment are alleviating patient symptoms, such as tremors and emotional sleep disturbances, and improving mobility. The clinical and experimental therapeutic strategies for SCA3 are now aimed at directly targeting polyQ proteins or preventing their downstream toxic effects. Recent studies have proposed gene suppression strategies to interfere with or suppress mRNA expression of the abnormal SCA3 gene with RNA interference (RNAi) and antisense oligonucleotides (ASOs) for SCA3 therapy [2,3,4]. Other potential therapeutic strategies include neurotransmitter modulators, autophagy enhancers, ion channel inhibitors, growth factors, and stem cell therapies [5]. Among these molecules, neuroprotective molecules, such as growth hormone (GH), insulin-like growth factor-1 (IGF-1), and nerve growth factor, have been tested in SCA3 animal models and in humans through clinical trials and have displayed potential therapeutic effects in patients with SCA3 [6,7].

Our recent study showed that GH treatment in SCA3 84Q transgenic mice restored locomotor functions, as indicated by the results of the rotarod test and box behavior analysis after 9 months, while reduced DNA damage and increased preservation of Purkinje fibers were also observed [8]. However, the underlying regulatory pathways and mechanisms by which GH ameliorates SCA-associated behavioral and neuropathological abnormalities are not fully understood. GH activates the JAK2 tyrosine kinase signaling pathway, which continues to activate multiple downstream pathways to promote cellular growth and regulate metabolism [9,10]. GH also promotes insulin-like growth factor-1 (IGF-I) to further growth-stimulating effects as Igf1 is a main transcriptional target of GH in different tissues [11,12,13]. Because SCA3 patients exhibit low glucose utilization, perturbed fatty acid and amino acid metabolism, and insulation signaling deficiency [5,14], GH or IGF-1 therapy may modulate metabolism that is reprogrammed during the pathological development of SCA3. However, the serum growth hormone concentrations determined with radioimmunoassay did not show differences between SCA3 transgenic mice (Q71 homozygotes) and wild-type mice [15]. Altered serum levels of insulin and insulin-like growth factor 1 system were reported in patients with SCA3, with lower concentrations of insulin and insulin binding protein 1 (IGFBP1) and higher levels of insulin binding protein 3 (IGFBP3) [16]. Patients with late-onset cerebellar ataxia were reported to have lower serum IGF-1 levels [17]. In addition, the assessment of rating of ataxia scores (SARAs) was improved in patients with SCA3 in open-label trials of IGF-1 therapy for autosomal dominant cerebellar ataxia patients [7,18]. These findings suggest that the IGF-1 system is a potential player in SCA3 pathogenesis. We thus aimed to investigate the underlying mechanisms and the biomarker profile in the IGF-1 system by computational analysis of transgenic mice omics data.

To further dissect the pathological changes in SCA3 and potential therapeutic strategies, in the present study, we performed RNA-seq to analyze the transcriptomic profile of SCA3 84Q transgenic mice compared to control 15Q mice as well as SCA3 84Q mice treated with IGF-1. Metabolic network models of mice with SCA3 were constructed based on the genome-wide RNA-seq data to explore the metabolic alterations in SCA3. The SCA3 84Q group showed decreased glycolytic flux, fatty acid degradation, and ATP production through OXPHOS (oxidative phosphorylation). The IGF-1-treated SCA3 84Q group showed increases in OXPHOS ATP production and glucose metabolism. The changes in differentially expressed (DE) genes in the IGF-1 groups indicated the activation of cell growth and metabolism-related signaling pathways. The proposed SCA3 genome-scale metabolic model provides novel ways to analyze the important biochemical pathways and identify potential biomarkers in SCA3.

## 2. Results

### 2.1. Exploratory Data Analysis

**Figure 1:**
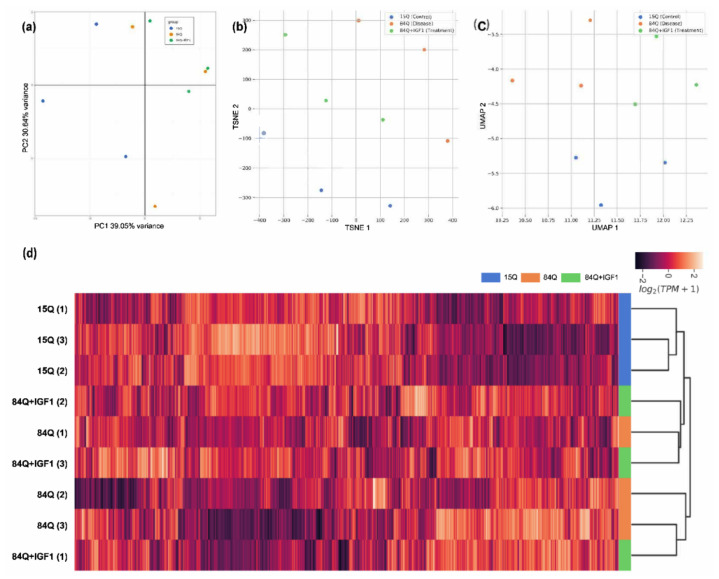
Exploratory analysis of the RNA-seq data in the SCA3 transgenic mouse model. Raw counts were log-transformed into the log2 scale and were analyzed with principal component analysis (PCA): (**a**) Transcript per kilobase million (TPM) values were analyzed and visualized via t-SNE; (**b**) UMAP; (**c**) and hierarchical clustering; (**d**) according to the modified log2 transformed TPM values, where standard deviations among the samples were greater than 3. The transformed values were further transformed into z-scores (by column). Blue indicates the control group (15Q), red indicates the 84Q sham group (84Q), and green indicates the IGF-1-treated 84Q group (84Q+ IGF-1).

Dimensional reduction was applied to analyze gene expression data to provide insight into the association between samples. Principal component analysis (PCA) of the rlog-normalized gene counts from mRNA gene expression data revealed clear differences between the control group (15Q) and the SCA3 sham group (84Q), but the IGF-1 treatment group (84Q+IGF-1) did not show clear differences from the 84Q group (Figure 1a). The respective genotype clusters were moderately separated along with the 1st principal component (PC), which explained 39% of the variance in the genotype, and along with the 2nd PC, which explained 31% of the variance. Similar trends were also observed in t-distributed stochastic neighbor embedding (t-SNE) (Figure 1b), uniform manifold approximation and projection (UMAP) (Figure 1c), and hierarchical clustering analysis (Figure 1d) in transcripts per kilobase million (TPM).

### 2.2. Differential Gene Expression (DGE) Analysis

**Figure 2:**
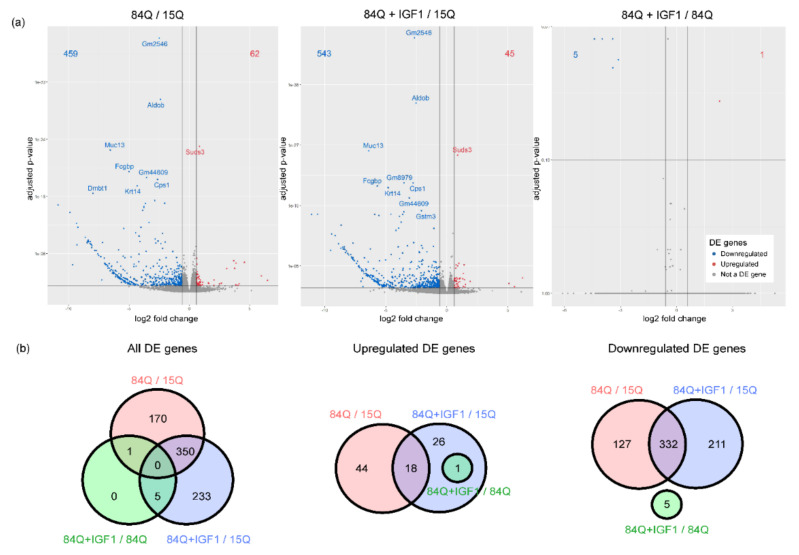
Differential gene expression analysis: (**a**) A volcano plot using the average fold-change (FC) and adjusted *p*-value to illustrate genes with significant differences. The red dots are upregulated genes with log2(FC) > log2(1.5); the blue dots are downregulated genes with log2(FC) < −log2(1.5). (**b**) Venn diagrams of differentially expressed (DE) genes that are altered (left), had downregulated expression (middle), or upregulated expression (right) between 15Q and 84Q, 15Q and 84Q+IGF-1, and 84Q and 84Q+IGF-1, respectively.

To further assess the pathological features of SCA3-84Q, we compared the global gene expression profiles of the 15Q group and the SCA3-84Q group. Compared with those in the 15Q group, 521 genes were differentially expressed in the 84Q group, with an adjusted *p*-value < 0.05 (62 genes with upregulated expression and 459 genes with downregulated expression); additionally, 588 genes were differentially expressed in SCA3-84Q mice with IGF-1 treatment (45 genes with upregulated expression and 543 genes with downregulated expression). Comparing SCA3-84Q with IGF-1 to SCA3-84Q, there were only 6 differentially expressed genes, 5 of which (ribosomal proteins Rps8-ps1, Rps8-ps4, and Rps8-ps2, and pseudogenes Gm14414 and Gm14438) were downregulated and 1 of which (calpain 11 protein, Capn11) was upregulated, as shown in the volcano plot (Figure 2a). A subset of 350 genes was differentially expressed in both 84Q vs. 15Q and 84Q+IGF-1 vs. 15Q comparisons as shown in the Venn diagram (Figure 2b), including Suds3 (SIN3A corepressor complex component), which was upregulated in both comparisons; and Aldob (Fructose-1,6-bisphosphate aldolase), Cps1 (carbamoyl-phosphate synthase 1), Fcgbp (Fc fragment of IgG binding protein), Krt14(keratin 14), Muc13(mucin 13, cell surface-associated), and Gm2546(glyceraldehyde-3-phosphate dehydrogenase pseudogene), which were downregulated in both comparisons.

### 2.3. Functional Enrichment Analysis

**Figure 3:**
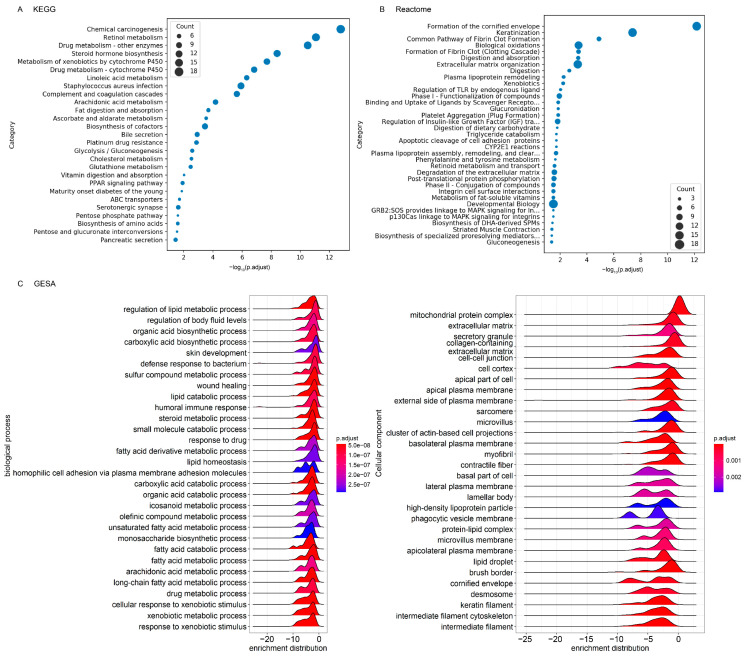
Scatter plot of enriched biological pathways comparing control-15Q to SCA3-84Q. The horizontal axis represents the enriched: (**A**) KEGG and; (**B**) Reactome pathways. The vertical axis represents the gene ratio of each pathway. The gene ratio refers to the ratio of the number of enriched DE genes to the number of annotated genes. The pathway was listed when the BH-adjusted *p*-values were lower than 0.05 and the *q*-values were lower than 0.2 in the hypergeometric tests; (**C**) Gene set enrichment analysis (GSEA) based on GO term annotations of biological processes and cellular components.

In functional enrichment analysis, gene ontology (GO) annotates genes to biological processes, molecular functions, and cellular components, while the Kyoto Encyclopedia of Genes and Genomes (KEGG) and Reactome annotate genes to pathways and reaction networks. Two hundred and fifty-five significantly enriched GO terms in the 84Q group compared with the 15Q group were identified (Table 1 and Table S1), with 22 related to cellular components, 49 related to molecular functions, and 184 related to biological processes with BH-adjusted *p*-value < 0.01. The molecular function category contained glycosaminoglycan-binding, carboxylic acid-binding, heparin-binding, steroid hydroxylase activity, and oxidoreductase activity. The biological process category contained fatty acid metabolic process, steroid metabolic process, xenobiotic metabolic process, drug metabolism, arachidonic acid metabolic process, extracellular structure organization, intermediate filament organization, and coagulation. Consistent with these findings, the gene set enrichment analysis highlighted significantly altered biological processes in lipid metabolism, xenobiotic metabolism, and cellular components in an extracellular matrix organization (Figure 3C).

However, only one molecular function related to cysteine-type endopeptidase activity was identified as enriched GO terms in SCA3-84Q with IGF-1 compared to SCA3-84Q, which are involved in apoptotic signaling pathways.

Twenty-seven significantly enriched KEGG pathways and twenty-four Reactome pathways in the 84Q group compared with the 15Q group were identified (Figure 3 and Tables S2 and S3). These pathways involved fatty acid biosynthesis and metabolism (including steroid hormone biosynthesis, arachidonic acid metabolism, linoleic acid metabolism, and triglyceride catabolism), glutathione metabolism, amino acid biosynthesis, biological oxidation, carbohydrate metabolism (including carbohydrate digestion and absorption, and ascorbate and aldarate metabolism), and retinol metabolism. Pathways associated with immune system diseases, such as type 1 diabetes mellitus, drug metabolism, chemical carcinogenesis, fibrin clot formation, serotonergic synapses, and infection and immune response were also identified. In summary, functionality analyses showed that several essential metabolic pathways, endopeptidase activity, and signaling pathways were decreased in the 84Q group compared to the 15Q group.

**Figure 4:**
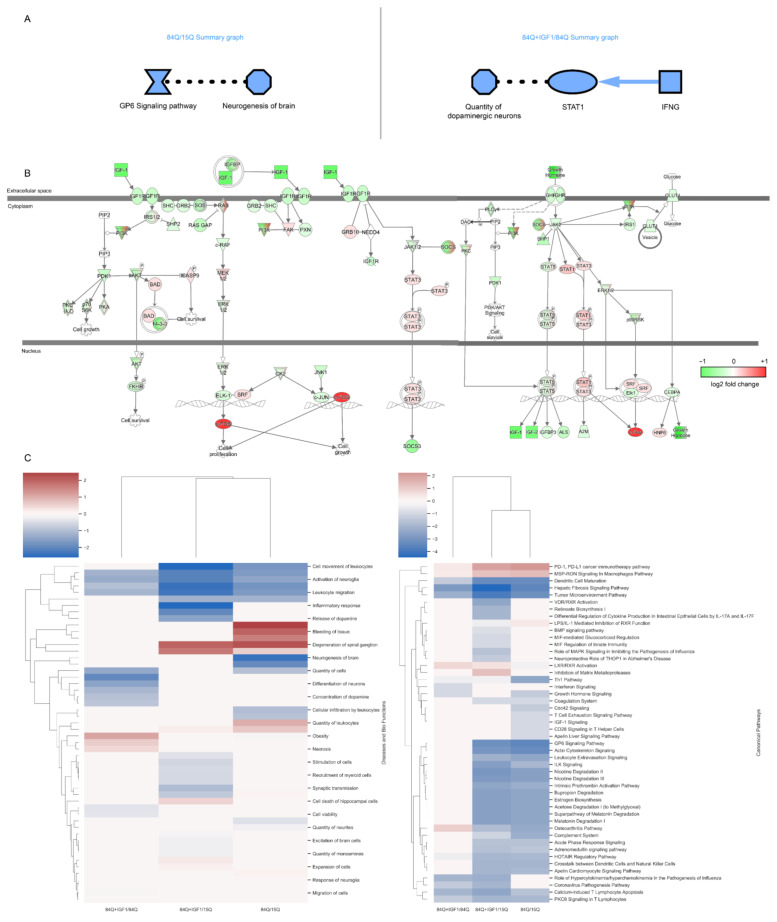
Ingenuity pathway analysis: (**A**) Graphical summary of the Ingenuity Pathway Analysis (IPA) core analysis for the 84Q and 84Q–IGF-1 groups. IPA core analysis, including canonical pathways, upstream regulators, and functions/diseases, graphically shows that neurodegeneration and cell death of neural cells are major events in the 84Q group. Red: significantly increased, green: significantly decreased, orange symbol and arrows: activation, blue symbol and arrows: inhibition, solid line: direct effect, and dashed line: indirect effect; (**B**) In the network analysis of DE genes in the 84Q group, the GH¬–IGF signaling pathway was identified as one of the significantly changed pathways in the 84Q group compared to those in the 15Q group by IPA. All the upregulated DE genes are mapped in red, while downregulated DE genes are shown in green. Members of the GH–IGF signaling family are either up- or downregulated; (**C**) IPA comparison of canonical pathways, and diseases and biofunctions between subgroups. For each function, z-scores were used to predict activation or inhibition.

In Ingenuity Pathway Analysis (IPA), 608 genes with upregulated expression and 1832 genes with downregulated expression in the 84Q group compared with the 15Q group and 767 genes with upregulated expression and 830 genes with downregulated expression in the 84Q–IGF-1 group compared with the 84Q group were used to identify overrepresented canonical pathways. Core analysis using IPA software indicated that when comparing the 84Q group to the 15Q group, the GP6 signaling pathway was strongly reduced and indirectly related to the decreased neurogenesis in the brain. In a comparison of the 84Q–IGF-1-treated group to the 84Q sham group, the number of dopaminergic neurons was decreased, and the inflammatory IFNG-STAT1 pathway was decreased (Figure 4A). Downregulated networks were identified around the insulin-like growth factor-1 (IGF-1) genes in the 84Q mice, where gene expression fold changes were mapped to the IGF-1 pathway (Figure 4B). A total of 34 and 16 enriched canonical pathways were identified by applying −log (*p*-value) > 2 thresholds in comparing the 84Q group to the 15Q group and the 84Q–IGF-1 group to the 84Q group, respectively. Most of the significantly enriched pathways were found to be tightly associated with the signaling pathways as shown in Figure 4C. Most of these signaling pathways, including the glycoprotein VI (GP6) signaling pathway, acute phase response signaling, integrin-linked kinase (ILK) signaling, intrinsic prothrombin activation pathway, growth hormone signaling, and apelin liver signaling pathway, were enriched by downregulated genes in the SCA3-84Q group. The complete list of enriched canonical pathways is included in Table S5.

The IPA also revealed potential biomarkers. Among the identified biomarkers, 40 were found in both the 84Q/15Q and 84Q–IGF-1/84Q comparisons, 27 of which had different gene regulatory directionalities in these two-group comparisons. IGF-1 was identified as one of the biomarkers in IPA with an expression log fold change of −0.671 along with 120 other molecules (Supplementary Table S6). A large portion of these differentially expressed genes are microRNAs (e.g., mir-17, mir-25, mir-28, mir-103, mir-132, mir-150, mir-154, mir-181, mir-345, etc.). microRNAs are associated with neurodegenerative diseases and SCA3 [19,20,21] and are potential biomarkers for SCA3 due to their stability in blood. Other differentially expressed genes were associated with cellular growth and differentiation (e.g., DLK1, FOS, and LTF), transcription (e.g., JUNB and NR4A1), and immune response (e.g., CCL2), indicating perturbations of cell growth and differentiation and immune response pathways in the 84Q group and the 84Q–IGF-1-treated group.

### 2.4. Metabolic Network Analysis

**Figure 5:**
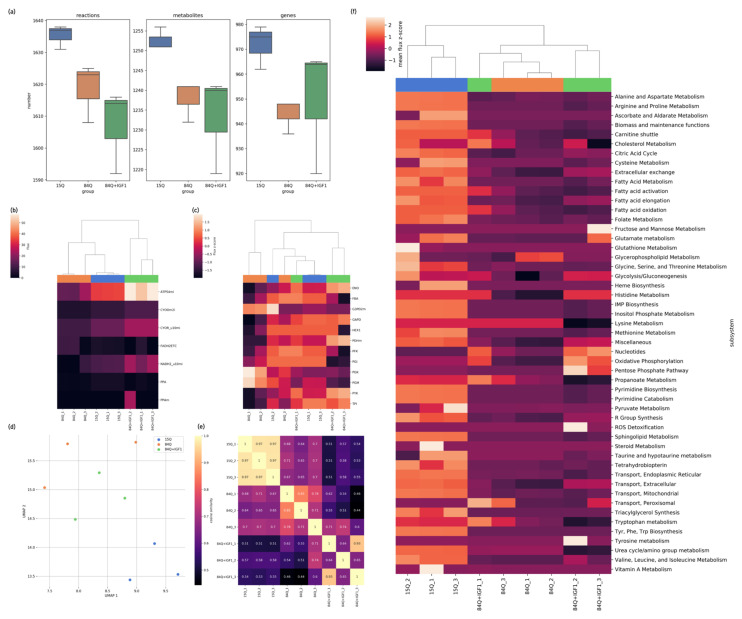
Metabolic network analysis: (**a**) Statistics of components in the constructed genome-scale metabolic network models (GEMs). The GEM was constructed individually from the RNA-seq data for each mouse from the 15Q, 84Q, and 84Q+IGF-1 groups; (**b**,**c**) Hierarchical clustering of fluxes from parsimonious flux balance analysis (pFBA), according to the metabolic subsystem oxidative phosphorylation (OXPHOS) and glycolysis. The fluxes were sums of fluxes in the subsystem, categorized according to their metabolic functions; (**d**) UMAP plot of predicted pFBA fluxes; (**e**) Heatmap of predicted pFBA flux correlation coefficient values in cosine similarity between context-specific GEMs. The higher the values are, the more consistent the two models’ fluxes are; (**f**) Heatmap of predicted pFBA fluxes in predefined biological subsystems in the GEMs. Blue represents the 15Q group, red represents the 84Q group, and the green represents the 84Q+IGF-1 group.

The constructed genome-scale metabolic network model based on iMM1415 and reduced to the final SCA3 mouse brain-specific genome-scale metabolic (GEM) models consisted of approximately 1600 reactions, 1200 metabolites, 950 unique metabolic genes, 5 compartments, and 10 subsystems (Figure 5a). There were more active genes specifically in the control 15Q group and more inactive genes specifically in the 84Q and IGF-1-treated groups. The numbers of active reactions and metabolites were also lower in the 84Q and 84Q–IGF-1 groups. The E-flux algorithm [22] was applied to constrain the flux lower and upper bounds by directly mapping normalized gene expression levels.

**Figure 6:**
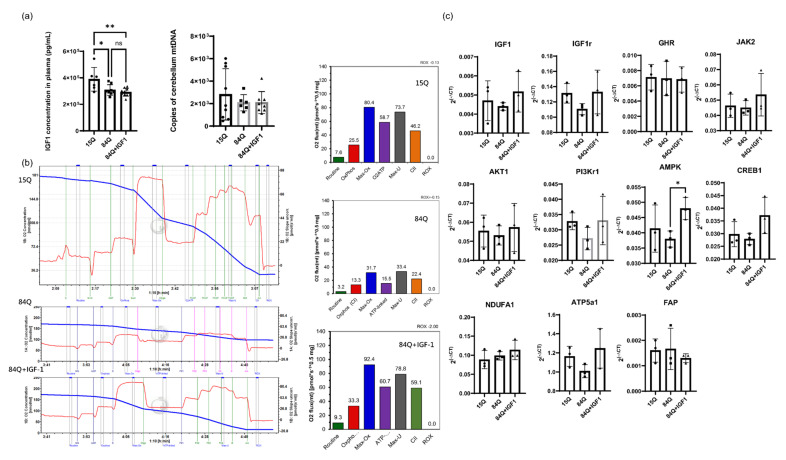
(**a**) IGF-1 concentration in plasma and mitochondrial DNA (mtDNA) copy number measured from the cerebellum of SCA3 mice. (**b**) Respiratory profile of cerebellar tissue. Respiration of tissue homogenate was measured by high-resolution respirometry (O2k; Oroboros Oxygraph-2k, Innsbruck, Austria) using substrate-uncoupler-inhibitor titration (SUIT) protocols [23], with modifications. Respiratory flux was expressed per milligram of protein, and the oxygen background fluxes were calibrated and subtracted from the total volume-specific oxygen flux (*n* = 2). (**c**) Validation of gene expression with qPCR. RNA-seq results were validated on the same RNA samples using qPCR. The selected genes tested were IGF-1, IGF-1r, GHR, AKT1, JAK2, PI3Kr1, AMPK, CREB1, NDUFA1, ATP5a1, and FAP. The mouse reference gene used was beta-actin (Actb) (*n* = 3, * *p* < 0.05, ** *p* < 0.01).

In the metabolic network analysis, pFBA was performed to simulate the flow of metabolites in the metabolic network in the 15Q, 84Q, and 84Q+IGF-1 groups. pFBA identified altered metabolic pathways and which components changed mitochondrial ATP production. Fluxes simulated from the pFBA were categorized, normalized, and summed to yield the total flux in each metabolic subsystem group. The overall metabolic flux profiles were clustered into three groups (15Q, 84Q, and 84Q+IGF-1) by UMAP analysis (Figure 5d) with a low correlation coefficient in cosine similarity (Figure 5e). The reactions were classified into secondary metabolic pathways according to annotation in the mouse model, and the flux heatmap of these reactions and clusters was plotted (Figure 5f), showing that glycolysis, oxidative phosphorylation, fatty acid metabolism, sphingolipid metabolism, triacylglycerol synthesis, cholesterol metabolism, the pentose phosphate pathway, urea cycle/amino group metabolism, and some amino acid metabolic pathways (e.g., histidine metabolism) were decreased in the 84Q group. Glycolysis, oxidative phosphorylation, the citric acid cycle, nucleotides, and histidine metabolism were enhanced in the 84Q+IFG-1 group. The oxygen exchange flux was shown to decrease in the 84Q group compared to the control group and was recovered in the 84Q+IGF-1 group, as validated by the OCR measured by O2K high-resolution respirometry (Figure 6b). Fluxes in propanoate metabolism, tryptophan metabolism, lysine metabolism, and urea cycle/amino group metabolism were decreased in the 84Q+IGF-1 group.

**Figure 7:**
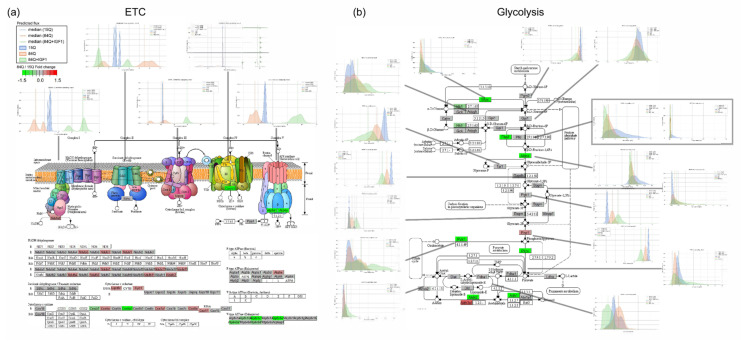
Flux sampling distributions of key reactions in the core metabolic pathways of the (**a**) electron transport chain (ETC) and (**b**) glycolysis. In the random sampling flux plot, green represents the 15Q group, red represents the 84Q group, and orange represents the 84Q+IGF-1 group. The upregulated DE genes are mapped to the metabolic pathways in red, while downregulated DE genes are shown in green based on the fold change for 84Q/15Q.

Sensitivity analysis, such as flux variability analysis and random sampling, evaluates ranges of fluxes through individual reactions in a network and determines the flexibility of metabolic networks to satisfy specific objective functions. Flux sampling results were plotted with the gene expression count in the electron transport chain pathway, glycolysis, and fatty acid metabolism. Glycolysis and ETC fluxes were decreased in the 84Q group and enhanced by IGF-1 treatment (Figure 5b,c and Figure 7). Interestingly, while respiratory flux through complex I in the mitochondrial electron transport chain was decreased in the 84Q group, the fluxes through complex II respiration and glycerol-3-phosphate dehydrogenase were enhanced possibly due to compensation to maximize ATP production. In glucose metabolism, most of the fluxes were decreased in the 84Q group but with increasing fluxes of phosphoglycerate kinase and phosphoglycerate mutase. When there was a difference between the gene expression level and the calculated metabolic flux distribution, as shown in Figure 7, it might be due to translational regulation, post-transcriptional control, or other factors affecting protein expression levels or enzyme activities.

## 3. Discussion

RNA-seq technologies have resulted in a rapid increase in available omics datasets. RNA-seq data with global gene expression patterns can also account for all RNA transcripts that are translated into enzymes for a given pathway. Large-scale metabolic networks using these transcriptomic data can be constructed and analyzed. As a result, the current study provides a comprehensive transcriptome and metabolic pathway analysis and presents a novel metabolic network model for the SCA3 mouse model.

The RNA-seq and metabolic flux analyses revealed low mitochondrial oxidative phosphorylation (OXPHOS) activities and a metabolic shift from glycolysis to other metabolic pathways. In addition to metabolic pathway alteration, other signaling and regulatory pathways, such as inflammation, blood coagulation, and cell death, were also observed in the SCA3 84Q group by IPA and GO term analysis, which is consistent with findings in the literature [24,25,26].

Cellular metabolism is a cellular phenotype defined by a set of chemical reactions under specific conditions, and alterations in metabolite concentrations and reaction fluxes can be used to describe a cellular response to changing environmental or pathological situations. SCA3 84Q transgenic mice showed a metabolic signature of downregulated fluxes in glycolysis, OXPHOS, and lipolysis, which might explain the hypometabolism observed in the cerebellum from the [18F]-fluoro-deoxyglucose positron emission tomography (FDG-PET) imaging of SCA3 patients [27]. The pathway analysis of the transcriptome also identified that fatty acid, triacylglycerol and cholesterol metabolisms were altered in the 84Q group, which implies the potential use of lipid biomarkers for SCA3. Triacylglycerol, ceramides, sulfatides, and glycerophosphoserine were proposed as potential biomarkers of disease progression from a metabolomic study of SCA3 mice [28], and palmitoleic acid (FFA 16:1) and linolenic acid (FFA 18:3) were identified from a metabolomic study of SCA3 patients’ serum samples [14]. 

Signaling pathways and transcriptional regulation are other aspects of alterations in the SCA3 mouse model and might be the cause or the effects of metabolic changes. In particular, the RNA-seq results showed that GH and IGF-1 signaling pathways were altered in the SCA3 84Q group, with lower expression levels observed in related pathways, which were confirmed by the IGF-1r qPCR results (Figure 6c). The IGF-1 concentration in the plasma was decreased in the 84Q group (Figure 6a), revealing that IGF-1 is involved in the pathological development of SCA3. GH exerts regulatory metabolic effects that increase free fatty acid (FFA) release and oxidation, decrease glucose and protein oxidation, increase protein synthesis, and decrease breakdown, leading to an increase in glycogen and lean body mass [29]. While IGF-1 acts downstream of GH as a central player in the somatotropic axis, IGF-1 activates mitogen-activated protein (MAP) kinase and PI3K signaling pathways to promote tissue growth and maturation through the upregulation of anabolic processes. The qPCR results quantitatively confirmed the expression changes in a few selected genes involved in the signaling pathway in GH–IGF-1 regulation. Among these genes, IGF-1r, PI3Kr1, and ATP5a1 levels were decreased in the pathogenic 84Q group, while GHR, AKT1, JAK2, FAP, and NDUFa1 levels were not (Figure 6c). However, IGF-1 treatment activated the expression of metabolic modulation-related genes, such as AMPK, and CREB, to compensate for the metabolic rewiring in the SCA3 disease state. These results confirm that the GH–IGF-1 pathway plays an important role in the development of SCA3. Therefore, serum IGF-1 and IGF-1 binding proteins could be potential biomarkers for SCA3 diagnosis.

**Figure 8:**
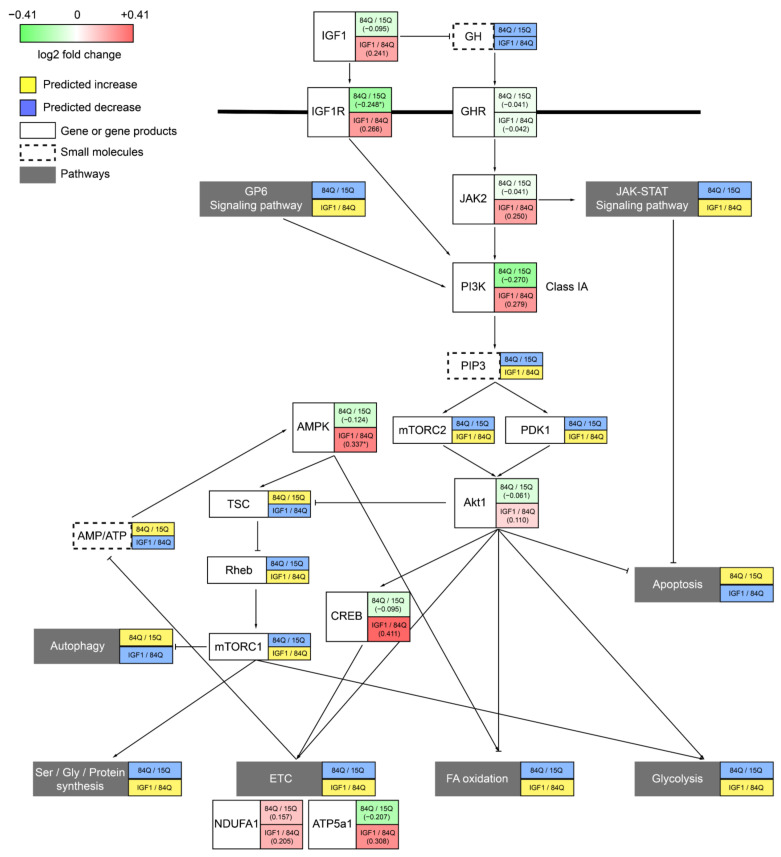
Summary of the IGF-1 and GH regulatory pathway downstream effects on brain metabolism and energetics. IGF-1 activates the PI3K pathway and AMPK and affects Akt1 pathways through mTORC and PDK1. Then pathways including cellular metabolism, mitochondrial function, and apoptosis are further regulated. Red: increase by IGF, green: decrease by IGF. The upper box shows the fold change for 84Q/15Q, and the lower box shows the fold change for 84Q+IGF-1/84Q.

Our previous study showed that GH has a benign effect on the SCA3 mouse model [8]. In the present study, the therapeutic effect of IGF-1 on SCA3 was explored by examining the transcriptomic and metabolic flux profiles in these transgenic mice. Regarding RNA expression and the calculated metabolic fluxes, the IGF-1 treatment group showed a trend toward recovery back to the control level, including mitochondrial oxidative phosphorylation and glycolysis flux. Although the IGF-1 neuromotor function correction to SCA3 84Q mice (unpublished data) was not as dramatic as that of 84Q mice with GH treatment (Figure S1), the metabolic features of the IGF-1-treated group became closer to those of the control 15Q group than to those of the pathological 84Q group, indicating IGF-1 affects cellular metabolism in SCA3. The expression levels of signaling molecules AMPK, JAK2, and CREB1 were even higher in the IGF-1-treated 84Q group. Figure 8 summarizes the gene expression fold changes from the qPCR analysis based on mapping to the major signaling pathway molecules involved in the GH–IGF-1 pathway. Notably, brain regions and age (disease stage) are factors affecting SCA3 gene expression and metabolic profiles [28]. Our RNA-seq sample was taken from the cerebellum with mixed regions, and IGF-1 was administered through intraperitoneal injections, which might reduce the treatment effect of IGF-1 in 84Q mice.

In summary, we describe the generation of tissue-specific diseases via an in silico metabolic model as an in vitro disease modeling platform. Transcriptional and metabolic profiling of SCA3 transgenic mice showed alterations in signature biochemical pathways and changes in biomarker molecules. Low glucose and fatty acid utilization, altered amino acid metabolism, and deficient IGF-1 signaling were revealed as pathological features of SCA3. Our study provides not only interaction networks between the genes and metabolic fluxes for understanding the biological properties but also useful pathway maps for future understanding of the disease and the identification of new therapeutic targets.

## 4. Materials and Methods

### 4.1. SCA3 Transgenic Mouse Model

Control mice with the YAC transgene expressing the human ATXN3 gene containing a polyglutamine tract with 15 CAG repeats (15Q) were donated by Dr. Henry L. Paulson’s laboratory [30]. SCA3 transgenic mice with an allele containing a pathological polyglutamine tract with 84 expanded CAG (84Q) repeats were obtained from The Jackson Laboratory (stock number 012705, Bar Harbor, ME, USA). The 15Q and 84Q transgenic mice were administered intraperitoneal injections of PBS (0.01 M) or IGF-1 (50 mg/kg) weekly from the postnatal age of 9 months to 18 months [8] and are denoted as 15Q (control group), 84Q (SCA3 sham group), and 84Q+IGF-1 (IGF-1 treatment group). All animal experiments were approved and followed the guidelines of the Animal Care and Use Committee of Changhua Christian Hospital (CCH-AE-106-017).

Mouse tail tissue genotyping was performed after weaning newborn mice, and PCR primers were designed to amplify human ATXN3 gene fragments (forward primer 5′ TGGCCTTTCACATGGATGTGAA and reverse primer 5′CCAGTGACTACTTTGATTCG). The endogenous housekeeping gene beta-globin was used as the internal control (forward prime, 5′GTGCAACCATTGCCCTAAGT, and reverse primer 5′CAGCCAGCATCTCAGGTGTA). Blood samples were collected into microtubes containing EDTA and centrifuged at 2500 rpm for 15 min at room temperature. The concentrations of growth hormone and IGF-1 in the plasma were determined by enzyme-linked immunosorbent assays (ELISAs) using a Rat/Mouse Growth Hormone ELISA Kit (Merck Millipore, Billerica, MA, USA) and a Mouse/Rat IGF-I/IGF-1 Quantikine ELISA Kit (MG100, R&D Systems, Minneapolis, MN, USA), respectively. Fluorescence-based quantitative PCR (qPCR) was used to determine the mitochondrial DNA (mtDNA) copy number and gene expression levels in mouse cerebral tissue using the LightCycler-FastStart DNA Master SYBR Green I Kit (Roche Molecular Biochemicals, Pleasanton, CA, USA) as described previously [31].

### 4.2. RNA Extraction and qPCR

Total cerebellar RNA was extracted using TRIzol Reagent (Invitrogen, Carlsbad, CA, USA) according to the manufacturer’s instructions (Invitrogen, Carlsbad, CA, USA). The quantity and purity of RNA were determined through spectrophotometry by using a Nanodrop ND-1000 (Nanodrop Technologies, Wilmington, DE, USA). One microgram of total RNA was reverse transcribed using a Transcriptor First Strand cDNA Synthesis Kit (Roche Diagnostics, IN, USA) according to the manufacturer’s instructions and then stored at −80 °C.

Gene expression assays were designed using Roche Universal Probe Library (UPL) online application (https://lifescience.roche.com/en_tw/brands/universal-probe-library.html#assay-design-center, accessed on 10 November 2020). Primer sequences were verified using BLAST and Primer-BLAST analysis (NCBI) to ensure specificity (Table S8). β-actin was used as a reference gene (internal control) [21]. qPCR was performed in a total volume of 20 μL containing 10 μL of 2 × LightCycler 480 Probes Master mix, custom-designed forward and reverse primers (0.5 μM), unique UPL probe (0.1 μM, UPL set, Roche), and 2 μL of properly diluted cDNA and PCR grade distilled water. Thermal cycling was performed using a LightCycler 480 instrument (Roche) with the following cycling conditions: 95 °C for 10 min, followed by 45 cycles of 95 °C for 10 s and 60 °C for 30 s. qPCR was performed in duplicate. A blank control consisting of no template (in distilled water) was performed. The relative gene expression was calculated using the 2^−ΔCt^ method: ΔCt (sample) = (target gene Ct) − (β-actin Ct).

### 4.3. High-Resolution Respirometry

After sacrifice, approximately 10 mg of mouse cerebellum was immediately homogenized and suspended in 0.5 mL of miR05 buffer. The homogenized mouse cerebella were injected into a sealed Oroboros^®^ Oxygen-2K chamber (Oroboros Instruments, Innsbruck, Austria) at a final concentration of 2 mg/mL, and mitochondrial respiration was measured at 37 °C. This device can record the oxygen concentration in two parallel 2-mL chambers at the same time. According to the rate of change in the oxygen concentration, the oxygen flux of mitochondrial respiration was quantified. Subsequent titrations of substrates and inhibitors were sequentially added to the chambers to estimate mitochondrial oxygen consumption [32]. Malate (0.5 mM) and L-glutamate (10 mM) were evaluated to determine non-ATP-linked respiration (Routine). ADP (2.5 mM) was added to determine the oxidative phosphorylation capacity (OxPhos). The maximum oxidative capacity (Max-Ox) was determined by adding succinate (10 mM). Oligomycin (5 μM) was then added to inhibit ATP synthase to determine proton leak respiration (ATP-link). The uncoupler protonophore FCCP (1.5 μM) induced maximum uncoupled respiration (Max-U). The experiment was terminated by administering rotenone (10 μM) and antimycin A (6.25 μM) to inhibit complexes I and III, respectively, to obtain the residual oxygen respiration (ROX) to correct the respiratory oxidative capacity results.

### 4.4. RNA Sequencing of Mouse Cerebellar RNA

RNA libraries were constructed using an Agilent SureSelect Strand-Specific RNA Library Preparation Kit followed by size selection with AMPure XP beads (Beckman Coulter, Indianapolis, IN, USA). Libraries were sequenced as paired-end 150 bp reads on an Illumina sequencing platform (Illumina, San Diego, CA, USA) with a depth of ~20 million reads each. Sequencing data (FASTQ reads) were generated based on Illumina’s base-calling program bcl2fastq v2.20.

### 4.5. Preprocessing RNA-Seq Data

**Figure 9:**
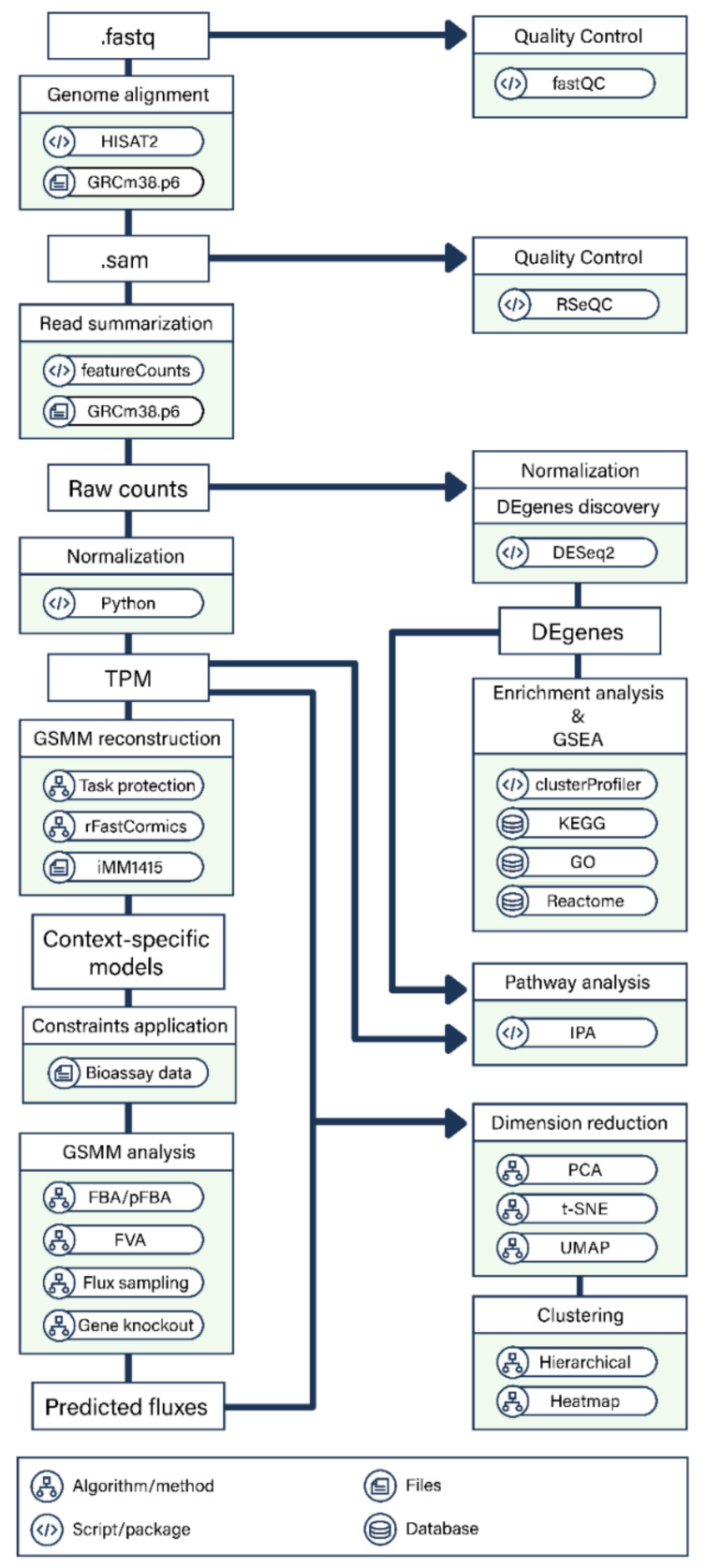
Overview of the transcriptomics analysis of the SCA3 transgenic mouse model.

After the samples were sequenced, a pipeline was used to analyze RNA sequences (Figure 9).

FastQC was first used for quality control of RNA-seq reads. Trimmomatic v0.36 [33] with the sliding-window approach was used for adaptor clipping and sequence quality trimming. HISAT2 [34] was used to map trimmed and filtered reads to the reference genome GRCm38.p6 (mm10) (https://www.ncbi.nlm.nih.gov/assembly/GCF_000001635.20/, accessed on 25 May 2020). The quality of the reads was reassessed with RSeQC after this step to confirm quality improvements. RNA-seq raw counts for each gene were generated using featureCounts [35], and the read counts were normalized to transcripts per kilobase million (TPM) by gene length and sequencing depth.

### 4.6. Expression Profile

Exploratory data analysis (EDA) summarizes the main characteristics of omics data and identifies potential batch effects and outliers. Dimension reduction analysis and clustering analysis are often performed in EDA.

Dimensionality reductions by principal component analysis (PCA), t-distributed stochastic neighbor embedding (t-SNE) [36], and uniform manifold approximation and projection (UMAP) [37,38] were used to visualize the RNA expression and metabolic flux profiles to evaluate component interactions. Briefly, PCA presents variations in the data with few variables using linear orthogonal transformation. t-SNE uses a Gaussian distance in high-dimensional space to analyze the similarity of points and projects these data into a low-dimensional space. UMAP estimates the topology of high-dimensional data and constructs a low-dimensional representation with preserved relationships. Clustering analysis uses the gene expression dimension to group different genes based on their similarities to discover hidden patterns at the systems level. The read counts and TPM values of each sample were analyzed and visualized by dimensional reduction analysis, hierarchical clustering, and heatmap production. Regularized log-transformation and PCA applied to read count data were conducted using the R package DESeq2 v1.12.3 [39]. The t-SNE and UMAP used on TPM data were implemented by Python package scikit-learn v0.24.2 [40]. Hierarchical clustering was performed and the heatmap was plotted using Seaborn v0.11.1 [41].

### 4.7. Differential Gene Expression (DGE) Analysis and Functional Enrichment Analysis

The package DESeq2 was used to detect DE genes from RNA-seq data. The Wald significance test was applied to identify the most significantly enriched pathways among the DE genes. A *p*-value adjustment method, the Benjamini and Hochberg (BH) procedure, was used to lower the false discovery rate (FDR) and to obtain the adjusted *p*-value of the test. Genes with ≥log2 (1.5) absolute log2-fold changes and adjusted *p*-values < 0.1 were considered significantly differentially expressed.

The DE genes of each experimental design were analyzed using clusterProfiler v3.6 [42]: Gene Ontology (GO) enrichment analysis [43], Kyoto Encyclopedia of Genes and Genomes (KEGG) pathway enrichment analysis [44], ReactomePA [45] for Reactome pathway enrichment analysis, and clusterProfiler for gene set enrichment analysis. KEGG pathways were visualized with RNA-seq data integrated using an R package, Pathview v1.30.1 [46]. A hypergeometric test was used in the analyses, and the pathways with a BH-adjusted *p*-value < 0.05 and a *q*-value < 0.1 were considered enriched pathways.

In addition to pathway enrichment analysis, gene set enrichment analysis (GSEA) was performed on the results obtained in the differential gene expression (DGE) analysis by leveraging gene sets provided by the GO, KEGG, and Reactome databases. In GSEA, the ranked list of genes was ordered by their log2-fold changes calculated by DESeq2. The permutation time was set to 1000 to generate the normalized enrichment score and calculate the FDR q value of each gene set. The FDR values of the selected results were less than 0.05.

Pathway analysis was performed to explore the canonical pathways, regulatory networks, and biomarkers, and the results were compared using the Ingenuity Pathway Analysis (IPA) system (QIAGEN Inc., https://www.qiagenbioinformatics.com/products/ingenuity-pathway-analysis as of 4 January 2021; version 60467501). Mean TPM (within-group) > 1 and |log2(FC)| > 0.5 were used to identify DE genes in IPA.

### 4.8. Metabolic Network Model Reconstruction and Flux Analysis

An SCA3 genome-scale metabolic (GEM) model was constructed based on the mouse model iMM1415 (http://bigg.ucsd.edu/models/iMM1415, accessed on 20 September 2020) [47], which contains thousands of reactions, metabolites, and transport processes derived from the human metabolic model Recon 1. The context-specific reconstruction algorithm FASTCORMICS RNA-seq workflow (rFASTCORMICS) was used to integrate RNA-seq data and construct the metabolic network [48]. rFASTCORMICS uses the gene expression intensity distribution across all genes to discretize the genes using Gaussian curves (expression curves) into three categories: expressed, unknown expression status, and unexpressed. The classified genes were then mapped to 3 sets of reactions, core reactions, noncore reactions, and inactive reactions, following the gene-protein-reaction rules (GPR rules). rFASTCORMICS is therefore specifically useful for analyzing sample-specific and tissue/cell-specific RNA-seq data without using arbitrary thresholds.

The functionality of the model was evaluated using 210 metabolic objective functions and tasks as the protection method as described by Richelle et al. [49]. E-Flux translates gene expression to maximum metabolic flux constraints for individual reactions [22]. The model fluxes were also constrained by the high-resolution respiration data from O2K respirometry (Figure 6b).

The metabolic fluxes of the mitochondrial networks were analyzed and simulated using parsimonious flux balance analysis (pFBA), flux variability analysis (FVA), and flux sampling. The optimization of the reaction fluxes was set to maximal production of the objective reaction. The biomass function and growth medium for the objective function were taken from the mouse metabolic model iMM1415 with no modifications. Metabolic network analysis was performed using COBRApy [50] and the linear programming solver Gurobi [51] in the Python environment. Other packages, including matplotlib, seaborn, scikit-learn, pandas, and NumPy, were also used to process and plot the results.

## Figures and Tables

**Table 1:** Selected gene ontology (GO) annotation of downregulated genes in SCA-3 transgenic mice. GO annotations were performed when the BH-adjusted *p*-values were lower than 0.05 and the *q*-values were lower than 0.2 in the hypergeometric tests. Fold changes were calculated by determining the ratio of the average normalized expression values between samples. The complete GO term list is in Table S1.

GO Term	Description	adj *p*-Value	Number of Genes
	**Cellular component**		
GO:0062023	collagen-containing extracellular matrix	2.41 × 10^−11^	31
GO:0016324	apical plasma membrane	1.34 × 10^−6^	22
GO:0005882	intermediate filament	7.64 × 10^−7^	14
GO:0031526	brush border membrane	1.34 × 10^−6^	11
GO:0045095	keratin filament	4.30 × 10^−8^	10
	**Biological process**		
GO:0006631	fatty acid metabolic process	2.81 × 10^−9^	29
GO:0042060	wound healing	2.81 × 10^−9^	27
GO:0008202	steroid metabolic process	4.43 × 10^−7^	22
GO:0043062	extracellular structure organization	1.09 × 10^−6^	21
GO:0006805	xenobiotic metabolic process	1.45 × 10^−12^	19
GO:0050817	coagulation	3.88 × 10^−6^	15
GO:0042737	drug catabolic process	1.09 × 10^−6^	10
GO:0045109	intermediate filament organization	4.18 × 10^−9^	10
GO:0019369	arachidonic acid metabolic process	3.62 × 10^−4^	8
	**Molecular function**		
GO:0005539	glycosaminoglycan binding	1.15 × 10^−6^	18
GO:0005201	extracellular matrix structural constituent	8.00 × 10^−8^	17
GO:0031406	carboxylic acid binding	7.20 × 10^−6^	17
GO:0008201	heparin binding	8.03 × 10^−6^	14
GO:0008395	steroid hydroxylase activity	1.85 × 10^−6^	11
GO:0016712	oxidoreductase activity, acting on paired donors, with incorporation or reduction of molecular oxygen, reduced flavin or flavoprotein as one donor, and incorporation of one atom of oxygen	5.75 × 10^−7^	11
GO:0016725	oxidoreductase activity, acting on CH or CH2 groups	6.20 × 10^−5^	5

## Data Availability

RNA-seq datasets analyzed in this study are available in the NCBI GEO repository, under accession number GSE178367.

## Supplementary Materials

The following are available online at https://www.mdpi.com/article/10.3390/ijms22157974/s1, Figure S1: IPA comparison analysis of canonical pathways between the 84Q treated with GH, 84Q sham, and 15Q control subgroups. Z-scores were used to predict activation (orange) or inhibition (blue). The 15Q and 84Q transgenic mice were administered intraperitoneal injections of PBS (0.01 M) or GH (0.05 mg/kg) weekly from the postnatal age of 9 months to 18 months (*n* = 2), Table S1: complete list from gene ontology annotation of upregulated and downregulated genes in SCA3-84Q transgenic mice compared to those in control-15Q mice, Table S2: DE genes in KEGG pathways, Table S3: Reactome analysis, Table S4: gene set enrichment analysis, Table S5: IPA canonical pathway analysis, Table S6: IPA biomarker discovery, Table S7: metabolic network model analysis, Table S8: Primer sequences used for qPCR.

## Abbreviations

BH	Benjamini and Hochberg procedure
DE gene	differentially expressed gene
FVA	flux variability analysis
GEM	genome-scale metabolic model
GO	gene ontology
GH	growth hormone
IGF-1	insulin-like growth factor-1
IPA	Ingenuity Pathway Analysis
KEGG	Kyoto Encyclopedia of Genes and Genomes
OXPHOS	oxidative phosphorylation
PCA	principal component analysis
pFBA	parsimonious flux balance analysis
SCA	Spinocerebellar ataxia
t-SNE	t-distributed stochastic neighbor embedding
TPM	transcripts per kilobase million
UMAP	uniform manifold approximation and projection
