# Supplementary material for: Transcriptomic and Metabolic Network Analysis of Metabolic Reprogramming and IGF-1 Modulation in SCA3 Transgenic Mice
Source: Int J Mol Sci. 2021 Jul 26;22(15):7974. doi: 10.3390/ijms22157974 (PMC8348158; doi:10.3390/ijms22157974)
Supplement: Supplementary file 1 [file ijms-22-07974-s001.zip › Supporting Information/Figure S1.pdf]

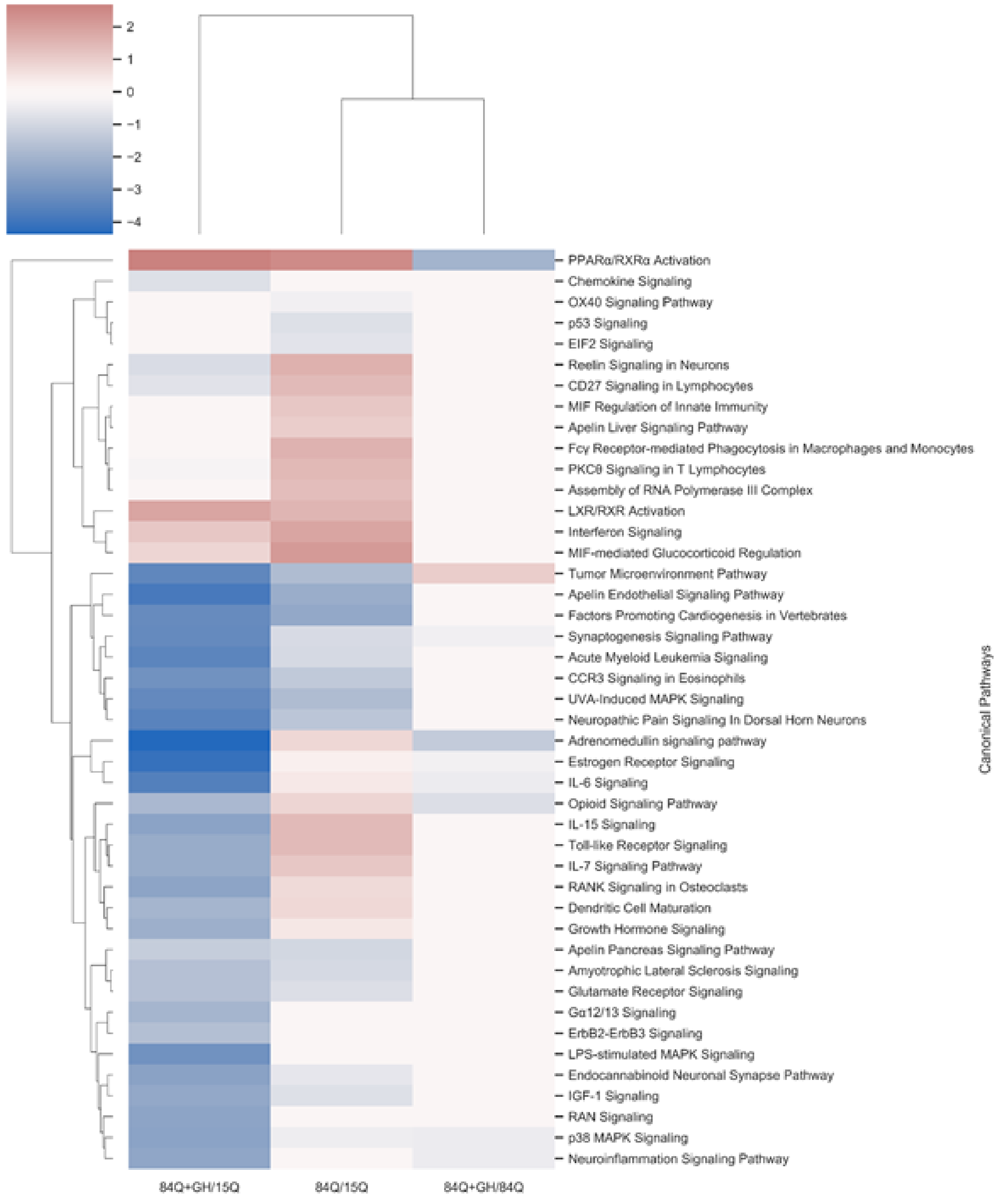

Figure S1.IPA comparison analysis of canonical pathways between the 84Q treated with GH, 84Q sham and 15Q control subgroups. Z-scores were used to predict activation (orange) or inhibition (blue) of each function. The SCA3 15Q and 84Q transgenic mice were administered intraperitoneal injections of PBS(0.01 M) or GH (0.05 mg/kg) weekly from the postnatal age of 9 months to 18 months (n=2). RNA libraries were sequenced as single-end 75 bp reads on an Illumina NGS System.
